# Whole-body vibration modulates leg muscle reflex and blood perfusion among people with chronic stroke: a randomized controlled crossover trial

**DOI:** 10.1038/s41598-020-58479-5

**Published:** 2020-01-30

**Authors:** Meizhen Huang, Tiev Miller, Michael Ying, Marco Y. C. Pang

**Affiliations:** 1 Department of Rehabilitation Sciences, The Hong Kong Polytechnic University, Kowloon, Hong Kong; 2 Department of Physical Therapy and Rehabilitation Science, University of Maryland School of Medicine, Maryland, United States; 3 Department of Health Technology and Informatics, The Hong Kong Polytechnic University, Kowloon, Hong Kong

**Keywords:** Rehabilitation, Stroke

## Abstract

This study aimed to investigate the acute effect of whole-body vibration (WBV) on the reflex and non-reflex components of spastic hypertonia and intramuscular blood perfusion among individuals with chronic stroke. Thirty-six people with chronic stroke (age: 61.4 ± 6.9 years) participated in this randomized controlled cross-over study. Each participant underwent two testing conditions: static standing for 5 minutes with WBV (30 Hz, 1.5 mm) or no-vibration. We assessed the soleus H-reflex, shear modulus (ultrasound elastography) and vascular index (color power Doppler ultrasound) of the medial gastrocnemius (MG) muscle on either paretic or non-paretic side at baseline and every 1-min post-intervention up to 5 minutes. The results revealed a significant inhibition of the H/M ratio bilaterally for the WBV condition (absolute change on paretic side: 0.61 ± 0.35, *p* = 0.001; non-paretic side: 0.34 ± 0.23, *p* = 0.001), but not the control condition. The inhibition of H-reflex was sustained up to 4 minutes and 3 minutes on the paretic and non-paretic side, respectively. The vascular index of MG muscle was significantly increased only for the WBV condition [paretic: from 0.55 ± 0.07 to 1.08 ± 0.18 (*p* = 0.001); non-paretic: from 0.82 ± 0.09 to 1.01 ± 0.13 (*p* < 0.001)], which lasted for 3 minutes and 5 minutes, respectively. No significant change of the shear modulus in the MG muscle was observed, regardless of the testing condition. Based on our results, WBV had an acute effect on modulating spastic hypertonia dominated by hyperreflexia in people with chronic stroke and facilitating greater intramuscular blood perfusion. No acute effect on passive muscle stiffness was observed.

## Introduction

Spastic hypertonia is common in leg extensors after stroke^1^ which may impose negative effects on mobility and balance and compromise daily life activities^1,2^. Although the primary origin of spasticity is impaired reflex function, changes in muscle mechanical properties also occur^2^. Evidence has suggested that spastic hypertonia has both a reflex component (e.g. hyperreflexia) and a non-reflex component (e.g. muscle passive stiffness)^2^.

Whole-body vibration (WBV), a treatment modality involving the delivery of mechanical stimuli to the lower limbs via a vibration platform, has the potential to manage spastic hypertonia^3,4^. WBV has been shown to inhibit the Hoffman reflex (H-reflex) in healthy athletes and young adults^5–9^, which is due to the presynaptic inhibition of Ia afferents^10^, and/or the depletion of neurotransmitters within the presynaptic terminals^8^. Evidence also suggested that for young adults, WBV could produce a training effect in muscle by increasing tissue oxygenation^6^, blood perfusion^11^, and intramuscular temperature^12^. Moreover, the repetitive mechanical stretching endured by muscles and tendons during the vibration may reduce soft tissue stiffness as indicated by the improved sit-and-reach performance previously observed in healthy populations^13^. Thus, collectively, WBV may have the potential to modulate the passive mechanical properties of muscle^13,14^.

However, there is a paucity of evidence regarding the effects of WBV on the reflex and non-reflex components of spastic hypertonia post-stroke^3^. A thorough mechanistic investigation into the physiological effects of WBV on spastic hypertonia may elucidate the potential benefits of this modality. An electrically evoked H-reflex, which bypasses the muscle spindles, may be advantageous in assessing the modulation of monosynaptic reflex activity^15^. Its amplitude is a valuable parameter to quantify the hyperreflexia^15^. To measure passive muscle stiffness, ultrasound elastography, which has been shown to be a valid and reliable tool^16^, would be used in this study.

This study, therefore, aimed to investigate the acute effects of WBV on the reflex (soleus H-reflex) and non-reflex (passive stiffness of MG muscle) components of spastic hypertonia in both the paretic and non-paretic limbs of individuals with chronic stroke. Moreover, we also evaluated the blood perfusion of the MG muscle using power Doppler ultrasound^11,17,18^, because any changes in circulation induced by WBV may influence mechanical properties of muscle^13,19^. It was hypothesized that (1) WBV would result in an inhibition of the H-reflex, decreased muscle passive stiffness and increased muscle blood perfusion in both paretic and non-paretic legs, and that (2) the changes in the aforementioned outcomes would differ between the paretic and non-paretic sides.

## Methods

### Study design

This was a single-blinded randomized controlled cross-over study to investigate the acute effect of WBV on H-reflex, leg muscle blood perfusion, and leg muscle passive stiffness. This design was chosen to reduce the effects of the confounding variables that may occur when comparing different subject groups^20^.

### Sample size calculation

The sample size estimation was based on evidence from previous studies investigating the acute effect of WBV on H-reflex in people with stroke^21^ and intramuscular blood perfusion in a young healthy population^11^ using G*Power^22^. Chan *et al*. demonstrated that WBV could significantly reduce the H/M ratio in both legs with medium to large effect sizes (Cohen’s d of 0.70–0.90)^21^. Kerschan-Schindl *et al*. found that WBV could significantly increase the intramuscular blood volume based on Newman’s score among young adults, with a large effect size (Cohen’s d of 1.00)^11^. Currently, no study has reported the effect of WBV on muscle passive stiffness. Thus, a conservative medium effect size (f = 0.25) was expected in this study. Based on ANOVA analysis, with an alpha value of 1%, power of 80% and an attrition rate of 10%, the minimum required sample size was estimated to be 36 participants for this study.

### Participants

People with chronic stroke were recruited from a stroke patient self-help organization in the local community using convenience sampling. A recruitment letter was sent to the organization to invite their members to participate in the study. Those who indicated interest in participating were screened according to the following eligibility criteria. The inclusion criteria were (1) men or women at 18 years of age or more with a diagnosis of a hemispheric stroke >6 months, (2) medically stable, (3) able to stand independently for at least 1 minute and (4) Modified Ashworth Scale (MAS) score ≥ 1 measured at the ankle plantar flexors. The exclusion criteria were: (1) brainstem or cerebellar stroke, (2) other neurological condition, (3) serious musculoskeletal or cardiovascular disease, (4) severe contracture of the ankle that the ankle cannot be put in the neutral position, (5) metal implants or recent fractures in the lower extremities or spine, (6) fresh skin wounds in lower extremities, especially popliteal fossa, and (7) other severe illnesses or contraindication to exercise. Those who fulfilled all eligibility criteria were then enrolled in the study. All the participants provided written informed consent before data collection.

The study was approved by the Human Subjects Ethics Sub-committee of the University (Application Number: HSEARS20161117007) and was registered on ClinicalTrials.gov (NCT03015545; date of registration: January 10, 2017). All experiments were performed in accordance with the Helsinki Declaration ethical principles for medical research.

### Experimental procedures

**Figure 1:**
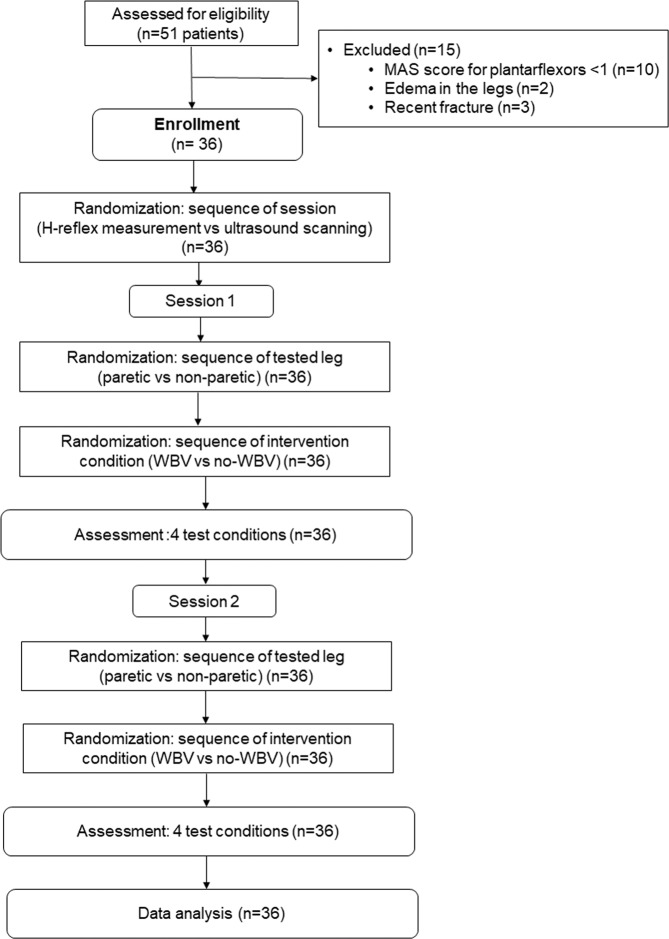
Experimental design. Each participant underwent two assessment sessions, one for soleus H-reflex measurement and the other for ultrasonographic scanning of the medial gastrocnemius muscle, in randomized sequence. For each session, the sequence of the side of the tested leg (paretic or non-paretic) was first randomized, followed by the randomization of the sequence of the intervention (with or without WBV). Thus, there were a total of four test conditions in each assessment session: (1) WBV intervention with measurement of the paretic leg; (2) non-WBV condition with measurement of the paretic leg; (3) WBV condition with measurement of the non-paretic leg; (4) non-WBV condition with measurement of the non-paretic leg. A 15-minutes wash-out period was applied between each test condition. A total of 36 participants with chronic stroke completed all assessment procedures.

All participants underwent two laboratory assessment sessions (one for recording the soleus H-reflex and another for the ultrasound scanning of the MG muscle). The two sessions were conducted in a randomized order by opening an opaque envelope containing a preassigned, computer-generated random sequence. Each session was separated by at least 24 hours but no more than 72 hours. During each session, all of the participants experienced four test conditions in a randomized sequence, generated by an online randomizer (www.random.org). The sequence of the side of the tested leg (paretic vs non-paretic) was determined first, following by the sequence of intervention (vibration vs no vibration). The randomization procedures are illustrated in Fig. 1, whereas the results of the randomization are shown in Table 1.

**Table 1:** Results of randomization (36 participants).

Sequence*	Number of participants			
	H-reflex measurement		Ultrasound scanning	
**a. Randomization of the testing session**				
Session 1	17		19	
Session 2	19		17	
**Sequence**^**†**^	**Number of participants**			
	**WBV, paretic leg**	**WBV, non-paretic leg**	**Non-WBV, paretic leg**	**Non-WBV, non-paretic leg**
**b. Randomization of test conditions of H-reflex measurement session**				
1^st^ test condition	11	6	7	12
2^nd^ test condition	7	10	9	10
3^rd^ test condition	8	13	12	3
4^th^ test condition	10	7	8	11
**c. Randomization of test conditions of ultrasound scanning session**				
1^st^ test condition	12	8	10	6
2^nd^ test condition	10	6	9	11
3^rd^ test condition	7	10	9	10
4^th^ test condition	7	12	8	9

*The two sessions were separated by at least 24 hours but no more than 72 hours.

^†^Each test condition was separated by a 15-minute rest period (washout).

**Figure 2:**
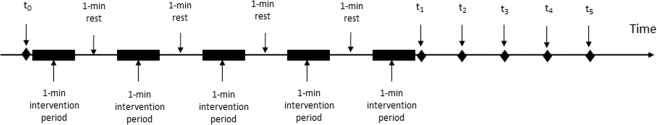
Measurement procedures for each test condition. Each test condition included five 1-minute bouts with at least 1 minute of rest between intervals. Measurements were taken immediately before the intervention (i.e., t_0_) and every minute after the termination of the intervention up to 5 minutes (i.e., t_1_, t_2_, t_3_, t_4_, t_5_).

For each test condition, the laboratory assessments were conducted immediately before the intervention (at t_0_) and each minute for 5 minutes after the termination of the intervention (t_1_, t_2_, t_3_, t_4_ and t_5_) (Fig. 2). Based on the results of our pilot trials and previous studies^8,23^, a minimum of 15-minutes of rest between each test condition was given to ensure the wash-out period was sufficient. Each assessment session lasted for approximately 2.5 hours and was conducted in the same university laboratory with the temperature kept constant at 25 °C.

Demographic information was obtained through patient interviews during the initial assessment. Motor function of the paretic leg was assessed using the Fugl-Meyer Assessment for the lower limbs, with higher scores indicating less impaired motor function^24^. Ankle plantar flexor spasticity was rated using the MAS, with a higher score indicating more severe spasticity^25^.

All of the measurements were performed by a researcher blinded to the intervention, while the intervention was facilitated by a different researcher not involved in the allocation and assessment of participants. The participants were required to refrain from strenuous exercise, stretching, alcohol, caffeine and any medications affecting the central nervous system for a period of 12 hours prior to the assessment^5^.

### Intervention

A synchronous vertical WBV platform (Fitvibe Excel, GymnaUniphy, Belgium) was used in this study. Participants were instructed to stand barefoot on the platform with knees flexed at 60° (i.e., in a squatting position) in both the WBV and control conditions. The WBV was set at a frequency of 30 Hz with an amplitude of 1.5 mm and validated with an accelerometer. For the control condition, the WBV device was turned off. Each condition consisted of five 1-minute bouts with at least 1-minute of rest between them (Fig. 2). During each trial, the participants were asked to place their feet parallel a shoulder’s width distance apart while distributing their body weight over each foot as evenly as possible. A researcher provided standby supervision to ensure that safety and correct posture were maintained. The exercise was terminated immediately if adverse symptoms (e.g., fatigue or dizziness) were reported. This intervention protocol was chosen because of several reasons. First, vertical vibrations were used rather than side-alternating vibrations. One potential disadvantage of using the vertical vibrations is the higher vibration transmission to the upper body^26^ and the lower degree of leg muscle activation^27^ compared with side-alternating vibrations. However, the major advantage of using the vertical WBV over side-alternating WBV is that the vertical WBV may pose less challenge to balance^27–29^ because it produces vertical displacements whereas side-alternating WBV generates both vertical and horizontal displacements^26–28,30,31^. Therefore, the added perturbations in both directions may make it more difficult for the participants to maintain balance while standing on the platform^27,28^. This is particularly relevant in individuals with stroke, because they often have some degree of balance deficits, including the suboptimal balance response to external perturbations^32^. Although such perturbations induced by the WBV platform may be useful in balance training^27,33^, it was not the objective of this study to improve balance ability through long-term exposure to WBV. Rather, it was more important for our participants to be able to maintain a static posture during the testing period. Therefore, after thorough consideration of the potential advantages and disadvantages, we decided to use synchronous vertical WBV in this study. Based on previous research on WBV, the vibration settings (e.g., type, frequency, amplitude) and body posture adopted here are considered appropriate and safe^34^ as the transmissibility of the vibration stimuli to the head is low enough to minimize discomfort (transmissibility <0.2) while the transmissibility to the legs is satisfactory to induce leg muscle responses (transmissibility >0.6)^35,36^. Second, five 1-minute WBV intervention periods were used here because similar protocols were shown to inhibit the H-reflex^6,8,9^ and facilitate the blood circulation in young healthy people^37^, and people with spinal cord injury^23^. Third, intermittent WBV periods would minimize the fatigue effect while the participants were in a deep squatting posture. This is particularly important for individuals with stroke, who may find it more difficult to sustain a particular posture for a long period of time and may fatigue more easily relative to their counterparts without stroke. There may be a higher risk of change in posture during testing if there was fatigue. Previous work has indeed shown that change in posture will greatly affect the vibration transmission in the body, which would be an important confounding factor^35^. Overall, the protocol used in this study was chosen after careful consideration, so as to ensure the quality of the data collection and participants’ safety.

### H-reflex measurement

The soleus H-reflex was measured using percutaneous stimulation of the tibial nerve while the participant assumed an erect standing position on the WBV platform^7^. After proper skin preparation, the receiving electrode (Nicolet Viking, Natus Medical Inc., Wisconsin, USA; 15 mm diameter) was positioned on the soleus muscle approximately 2 cm below the inferior margin of the two heads of the gastrocnemius muscle^5,6^. The reference electrode was placed at the base of the Achilles tendon insertion at the approximate level of the medial malleolus. The ground strap was placed over the proximal tibial head. All of the electrodes were connected to the Nicolet Viking Select electrodiagnostic system (Nicolet Viking, Natus Medical Inc., Wisconsin, USA). Calibrated surface impedances were lower than 5 kΩ. The signals were sampled at rates of 10 kHz, amplified by 1000, band-passed filtered between 2 Hz and 5 kHz and the sweep time was 5 ms per division^38^. A bipolar electrode was placed over the tibial nerve in the popliteal fossa^39^, and the site of stimulation was determined at baseline before any intervention was applied. Once the site was confirmed, the position of the probe was marked on the skin with semi-permanent pen to ensure a constant stimulation site for all trials.

During the stimulation, the participants were required to assume an erect standing posture and direct their gaze toward a fixed object placed at eye-level on the wall^15^. The H-reflex was elicited using a constant current with a square-wave pulse of 1 ms duration every 5 s while increasing the stimulus intensity by 0.1 mV until the maximum M wave (M_max_) of the soleus was determined^6^. Peak-to-peak amplitudes of the H-reflex and M-wave were automatically detected and calculated by a program (Nicolet Viking, Natus Medical Inc., Wisconsin, USA). The stimulation intensity of the H-reflex was then set at 10% of that required to evoke the M_max_. The M-wave was continuously monitored to ensure that the Ia afferents were excited to the same degree during each stimulation^8,40^.

For each test condition, three soleus H-reflex responses were evoked for each time point. Average H-reflex, M wave and H/M ratio values for each time point were computed. The magnitude of the H-reflex responses and M-wave were calculated as the means of the three responses at each time point for each participant. The H/M ratio was computed as the H-reflex response divided by the corresponding M-wave.

### Ultrasound measurement: muscle passive stiffness and blood perfusion

Muscle stiffness and blood perfusion of the bilateral MG muscle were assessed before and after WBV using an Aixplorer ultrasound scanner (Aixplorer Version 4.2; Supersonic Imagine, Aix-en-Provence, France) coupled with a linear transducer array (4–15 MHz, Super Linear 15-4; Supersonic Imagine, Aix-en-Provence, France).

As the elastic modulus of muscle (i.e., its stiffness) varies depending on joint position^41^, we carefully controlled the position of the ankle and knee joints during all assessments. The participants sat comfortably on a high-chair with the knee fully extended at 180° and the ankle fixed in a neutral position (i.e., at 90°) with a customized ankle stepping frame determined by the goniometer (Baseline HiRes Plastic 360 Degree ISOM, Fabrication Enterprises, White Plains, NY). This high sitting position was used rather than a prone position, because it was very challenging for stroke participants to change their position from a standing posture on the WBV platform to a prone position on a plinth in a short time. Thus, we chose to conduct the ultrasound scanning when the participants were sitting in a high chair, with strict control of the knee and ankle joint angles. Measurement points were marked on the skin to ensure that the ultrasound transducer was placed at the same site for all measures. Longitudinal and transverse points were marked at the proximal third of the tibial length between the crease of the popliteal fossa and the base of the Achilles tendon at the level of the medial malleolus^11,16^. Gel was applied between the transducer and the skin surface to minimize impedance and compression.

Muscle stiffness was measured along the fascicles of MG muscle in Supersonic Shear Imaging mode using the musculoskeletal preset of the Supersonic system. During the muscle stiffness measurement, the transducer was placed parallel to the muscle fascicles as confirmed by the transposed B-mode images^42^, because the transducer position in relation to the direction of fascicles may influence the elastography outcome^42^. Three images were captured within 10 seconds at every time point for each leg. The shear modulus of the MG muscle was measured over the region of interest (ROI) corresponding to the largest muscular region without visible intramuscular fascia^43^. The average value from the three images at each time point was calculated.

Intramuscular blood perfusion of the MG muscle was measured using the directional color power Doppler (dCPD) mode. The transducer was put in the transverse plane of the MG muscle. The size of dCPD color box was set to cover the entire cross-sectional area of the MG muscle. Doppler ultrasound settings were standardized for high sensitivity with a low wall filter to allow detection of vessels with low blood flow and low color noise^18^. The color gain was first increased to a level that showed color noise and then progressively decreased until the color noise was no longer apparent^18^. Color gain was kept constant for the same leg across all measurements. Three videos were captured at each time point for each leg. The vascular index (VI), a computerized method analogous to Newman’s grading scale, was used to quantify the intramuscular blood perfusion of the MG muscle^17^. The vascular index (VI) of the MG muscle was calculated as (the number of color pixels within the ROI)/(total number of pixels within the ROI) with larger values indicating greater blood perfusion^17^. Details of the image processing are provided in Supplementary 1.

Reliability testing of the ultrasound measurements was conducted in our pilot study. Fifteen individuals with stroke were measured twice after repositioning in the same session. The intraclass correlation coefficient (ICC) for the paretic and non-paretic MG muscle was 0.928 (95%CI = 0.887–0.959), and 0.932 (95%CI = 0.894–0.960) respectively.

### Statistical methods

All statistical analyses were conducted using IBM SPSS 22 (IBM, Armonk, NY). The dependent variables were soleus H/M ratio and the shear modulus and maximum vascular index (VI_max_) of the MG muscle. Data normality was inspected using the Shapiro-Wilk test. The baseline values (i.e., at t_0_) of all the dependent variables of the same leg were compared using paired-t-tests. To ensure that the M-wave did not change over time in response to the intervention, paired t-tests were conducted to compare M-waves across trials.

To test Hypothesis 1, two-way repeated measures ANOVA [within-subject factors: intervention (2 levels: with or without WBV) and time points (6 levels: t_0_~t_5_)] was used to compare each dependent variable for the paretic and the non-paretic legs. The results for the time × intervention interaction effect generated from the ANOVA models provide information about whether the changes in the outcome measures over time differed between the WBV and non-WBV conditions. When a significant time × intervention interaction was identified, post-hoc one-way repeated measures ANOVA analyses were conducted to examine the changes in outcomes over time for each experimental condition. To test Hypothesis 2, two-way repeated-measures ANOVA (within-subject factors: 2 sides and 6 time points) was used to compare each dependent variable between the paretic and non-paretic legs across time. In this ANOVA model, the time × side interaction provides information about whether the changes in outcome measures at each time point were different between the paretic and non-paretic sides. The sphericity assumptions of the ANOVA tests were verified using the Mauchly test. Greenhouse-Geisser adjustments were made if the sphericity assumptions were not met. Partial eta squared (ŋ_p_^2^) values of 0.14, 0.06 and 0.01 represent large, medium and small effect sizes, respectively^44^. A significance level of *p* ≤ 0.05 was set for two-way repeated ANOVA models, and a more stringent significance level of *p* ≤ 0.01 was set for post-hoc one-way repeated-measures ANOVA. Bonferroni correction for multiple comparisons was performed when appropriate.

## Results

Thirty-six participants with chronic stroke (mean age: 61.4 ± 6.9 years) completed all assessments and included in the analysis. No adverse effects were reported throughout the study. The demographic results are summarized in Table 2. There was no significant difference vascular index or shear modulus of the MG muscle for each leg at baseline, irrespective of testing conditions (*p* ≥ 0.05). The soleus H/M ratio was significantly higher on the paretic side than the non-paretic side (*p* = 0.013).

**Table 2:** Characteristics of participants (n = 36)*.

Variable^a^	Value		p
Age, year	61.4 ± 6.9		
Sex, men/women, n	26/10		
Body mass index, kg/m^2^	25.7 ± 3.2		
Total number of commodities, n^†^	2 (1–2)		
Total number of medications, n^†^	2 (1–4)		
Post-stroke duration, year	8.9 ± 5.0		
Type of stroke, hemorrhagic/ischemic, n	13/23		
Side of paresis, left/right, n	18/18		
	Paretic side	Non-paretic side	
Fugl-Meyer Lower limb motor score (full score: 34) ^†^	27 (25–29)	—	
Planterflexor Modified Ashworth Scale scores (range:0–5) ^†^	3 (2–4)	—	
Soleus H/M ratio	4.24 ± 2.24	2.17 ± 1.47	0.013^‡^
Vascular index of medial gastrocnemius	0.64 ± 0.40	0.82 ± 0.63	0.063
Shear modulus of medial gastrocnemius, kPa	25.05 ± 5.19	25.14 ± 4.95	0.298

*Mean (Standard deviation) indicated unless specified otherwise.

^†^Median (Interquartile range).

^‡^Significant difference between the paretic and non-paretic side (p < 0.05).

### Acute effect of WBV on H-reflex

**Figure 3:**
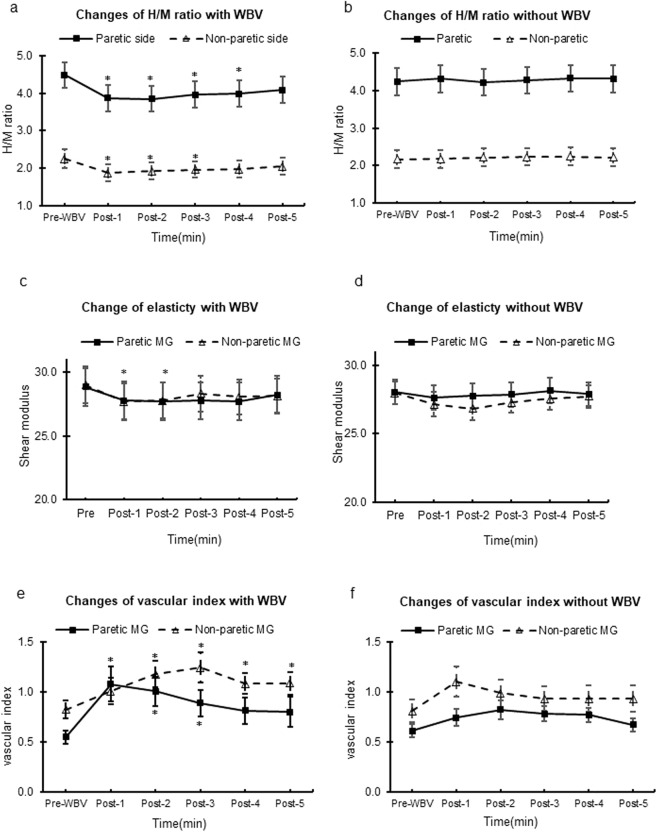
Acute effect of WBV. The soleus H-reflex, muscle stiffness and vascular index immediately before and every minute after the intervention. (**a**,**c**,**e**) Illustrate the measurement under the condition with WBV, and (**b**,**d**,**f**) Illustrate the measurement under the condition without WBV. *Significant difference compared with baseline (*p* < 0.01 with Bonferroni correction).

A significant time × intervention effect of soleus H/M ratio was found for both the paretic (*p* < 0.001, ŋ_p_^2^ = 0.234) and non-paretic (*p* < 0.001, ŋ_p_^2^ = 0.268) sides, indicating that the change in H/M ratio differed over time between the WBV and control conditions. On both sides, a significant change in H/M ratio over time was observed in the WBV condition (paretic side: *p* < 0.001, ŋ_p_^2^ = 0.249; non-paretic side: *p* < 0.001, ŋ_p_^2^ = 0.231) but not in the control condition (*p* ≥ 0.112). Post-hoc analysis showed that the H/M ratio decreased significantly after WBV exposure [H/M ratio absolute change on paretic side: mean = 0.61 (95%CI = 0.31 to 0.91), *p* = 0.001; non-paretic side: mean = 0.34 (95%CI = 0.21 to 0.46), *p* = 0.001] and inhibition of H-reflex activity was sustained for 4 minutes in the paretic leg and 3 minutes in the non-paretic leg. A significant time × side intervention was observed in the WBV condition (*p* < 0.001, ŋ_p_^2^ = 0.231), indicating that the absolute reduction in the H/M ratio was greater on the paretic side after the WBV intervention in comparison to the non-paretic side (Fig. 3a,b).

### Acute effect of WBV on muscle passive stiffness

The time × intervention effect of the shear modulus of the MG muscle was not significant for either the paretic or non-paretic sides (p = 0.307 and 0.513, respectively). The time × side effect was not significant for the WBV (p = 0.089) or control conditions (p = 0.400) (Fig. 3c,d).

### Acute effect of WBV on muscle blood perfusion

A significant time × intervention interaction effect for the vascular index of the MG muscle was observed in the non-paretic leg alone (*p* = 0.043, ŋ_p_^2^ = 0.071). The same interaction effect in the paretic leg was marginal (*p* = 0.066). A significant time effect was observed in the WBV condition for the paretic side (pre-WBV: 0.55 ± 0.07, 1-min post-WBV: 1.08 ± 0.18 *p* = 0.001, ŋ_p_^2^ = 0.105); and non-paretic side (pre-WBV: 0.82 ± 0.09, 1-min post-WBV: 1.01 ± 0.13, *p* < 0.001, ŋ_p_^2^ = 0.134), but not for the control condition (*p* ≥ 0.052). No significant time × side interaction was observed for either the WBV (*p* = 0.102) or control conditions (*p* = 0.180). Post-hoc analysis showed that the vascular index increased significantly after WBV exposure on both sides and was sustained for up to 3 minutes on the paretic side (*p* < *0.05*) and 5 minutes on the non-paretic side (*p* < *0.05*) (Fig. 3e,f).

## Discussion

The inhibition of the H-reflex was observed in the WBV condition but not the control condition. More specifically, the relative H/M ratio decreased by 14% and 17% from the baseline immediately the following vibration and was sustained for up to 4 minutes on the paretic side and 3 minutes on the non-paretic side. These results are in general agreement with those previously reported for healthy young adults^5–9^ and people with stroke^21^. These studies also reported an acute inhibition of the soleus H-reflex after WBV. However, the degree and duration of suppression ranged from 6% to 60% and from 1 minute to 20 minutes^5–9,21^. This may be due to methodological inconsistencies across studies with regards to WBV protocols and discrepancies in H-reflex measurement procedures such as participant positioning and electrical stimulation intensity^6,9^. In the current study, the H-reflex was measured in an erect standing position, which is a more functional position than others (e.g., sitting) for testing the soleus H-reflex and has important implications for standing balance and gait^6,15^.

The results indicated no significant changes in passive muscle stiffness after WBV exposure. Although WBV has been shown to increase muscle blood volume and metabolic rate^6,45^, leading to increased muscle temperature^12^, this change may be negligible. A previous study showed that after 10 minutes of WBV (frequency 26 Hz, amplitude 3 mm), muscle temperature increased by only 1 °C^46^. Subtle changes in temperature over a short period may not be sufficient to induce changes in the mechanical properties of muscle tissue^43^. Taken together, while the inhibition of H-reflex can be observed immediately after a very brief session of WBV, modulation of passive stiffness may require a longer WBV intervention period, which will need further investigation.

Previous research has found that WBV intervention of longer term may have the potential in reducing spastic hypertonia post-stroke. Ness *et al*. found that an eight-day WBV training (50 Hz, 2–4 mm) could significantly reduce quadriceps spastic hypertonia as measured by the pendulum test^47^. A randomized controlled trial by Pang *et al*. also provided preliminary evidence that 8-weeks of WBV training (20–30 Hz, 0.44–0.6 mm) reduced quadriceps MAS score in people with chronic stroke^48^. The relative contribution of the changes in reflex and non-reflex components to the reported improvement in spastic hypertonia after WBV training will require further study.

The vascular index increased on both the paretic and non-paretic sides following WBV but did not increase during the control condition. WBV has been shown to increase oxygen consumption without any substantial changes in blood pressure or heart rate among individuals with chronic stroke^45^. Furthermore, vibration may lead to increased shear stress in the vascular endothelium due to blood flow inertia, thereby promoting the release of endothelial-derived vasodilators such as nitric oxide^49^. Thus, increases in both metabolic demand and vasodilatory factors may influence muscle blood perfusion after WBV^49^. Comparable results have been observed in young healthy adults^6,11^ and people with spinal cord injury^23^. In current study, the vascular index of the MG muscle gradually increased twofold and was sustained for up to 3 minutes on the paretic side and 5 minutes on the non-paretic side. The difference in recovery duration between the paretic and non-paretic sides may be due to an altered vascular response to exercise and reduced arterial compliance in the paretic limb of chronic stroke survivors^50^. Nevertheless, these results demonstrate that the intramuscular perfusion of stroke survivors can be increased by WBV.

The participants in this study consisted of more men than women, with an average age of 61.4 years. Epidemiological studies showed that the occurrence of stroke was higher among people with advanced age and that the age-specific stroke rates were higher in men^51^. Therefore, our participants can be considered as quite representative of the overall stroke population, as far as sex and age are concerned. Based on previous research, the effects of age and sex on H-reflex amplitude were not significant^15^, although old age may have an impact on vasodilation function^52^. As this was a cross-over study, each participant served as their own control. Thus, the effect of confounding factors such as sex and age should be minimal. Nevertheless, the results of the study are only generalizable to those stroke patients who share similar demographic and clinical characteristics to our study participants.

This study may have important clinical implications. First, WBV produced an acute inhibitory effect on the H-reflex for stroke survivors, suggesting that it may have potential clinical applications in the management of spastic hypertonia. However, its long-term effect should be investigated in future studies. Second, soleus H-reflex inhibition was found to be associated with improved postural control in healthy adults^53,54^. A meta-analysis also revealed that WBV significantly improved balance among older adults^33^. Therefore, the potential association between H-reflex inhibition and gains in balance function induced by WBV warrants further research. Third, impaired blood perfusion is common in the paretic limbs after chronic stroke, which may contribute to metabolic dysfunction and functional decline^50^. Arterial remodeling and increased blood flow could be induced by resistance training^50^, while WBV was shown to have a comparable effect by increasing microcirculation in the skeletal muscles of young healthy adults^11,49^. This study also showed that a brief WBV session can increase intramuscular blood perfusion after stroke. Thus, WBV could be a useful training modality for increasing peripheral circulation. This is particularly relevant for frail stroke survivors, who are often unable to perform other forms of exercise. Future research should further elucidate the optimal WBV protocol for stroke patients with different disability levels.

This study has a few limitations. First, we only used a platform that generates synchronous vertical vibrations. It is worthwhile to replicate the study using side-alternating WBV in future research. Second, in this cross-over study, there was at least a 15-minute wash-out period between each intervention period. This had made the experimental sessions relatively long. However, the washout periods were important to minimize the effect of fatigue and carry-over effect from the previous intervention. While the factors arising from the lengthy data collection session (e.g., fatigue) could not be completely eliminated, the systematic influence on the results should be minimal because the sequence of testing was randomized. Third, the H-reflex was used as the main outcome measure rather than clinical tests such as the MAS, because MAS cannot differentiate between the reflex and non-reflex component of hypertonia, whereas the H-reflex specifically measures the reflex (neurological) component^55^. Moreover, MAS is a gross measure of hypertonia (i.e., on an ordinal scale) and is somewhat subjective, while the H-reflex measurement provides a more objective and precise estimate (i.e., being a continuous variable)^55^. Fourth, this study involved only a relatively small sample of stroke participants with mild to moderate motor impairments and a mean age of 61.4 years. The convenience sampling method used might have led to self-selection bias. For example, those who had more severe mobility deficits may not choose to join our study because of the need to travel to the university laboratory for assessment. The results of the study are only generalizable to those stroke patients who share similar characteristics to our study participants. Future work should involve a larger sample of stroke patients including those with more severe motor impairments. Nevertheless, as reflected by the MAS scores, our sample did include a wide range of individuals with different degrees of spastic hypertonia.

## Conclusion

Based on our results, WBV had an acute effect on modulating spastic hypertonia dominated by hyperreflexia in people with chronic stroke and facilitating intramuscular blood perfusion. An acute effect on muscle passive stiffness was not observed. Further work should use a larger sample size of stroke survivors with a wide spectrum of disability levels.

### Ethical approval

All procedures performed in studies involving human participants were in accordance with the ethical standards of the Human Subjects Ethics Sub-committee of The Hong Kong Polytechnic University (Application Number: HSEARS20161117007), and with the 1964 Helsinki declaration and its later amendments or comparable ethical standards

## Supplementary information

Supplementary Information
