# Supplementary material for: Whole-body vibration modulates leg muscle reflex and blood perfusion among people with chronic stroke: a randomized controlled crossover trial
Source: Sci Rep. 2020 Jan 30;10:1473. doi: 10.1038/s41598-020-58479-5 (PMC6992671; doi:10.1038/s41598-020-58479-5)
Supplement: Supplementary file 1 — Supplementary Information [file 41598_2020_58479_MOESM1_ESM.docx]

**Supplementary information for manuscript entitled**

Whole-body vibration modulates leg muscle reflex and blood perfusion among people with chronic stroke: a randomized controlled crossover trial

Meizhen Huang, Tiev Miller, Michael T.C. Ying, Marco Y.C. Pang

**Supplementary 1. Processing of vascular index.**

Data were processed using MATLAB2016a (Mathworks, Natick, MA, USA) with custom-written scripts. First, the dCPD videos were exported in “mp4” format from the Supersonic ultrasound system and sequenced in “jpeg” format. Next, the boundary of the MG muscle (i.e., the ROI) was manually outlined and extracted from the images. The total number of pixels in the ROI and the number of dCPD coded color pixels were then counted (Ying et al. 2009). The vascular index (VI) of the MG muscle was calculated as (the number of color pixels within the ROI)/(total number of pixels within the ROI) (Supplementary 4).(Ying et al. 2009) Finally, for each of the three videos, the three frames (i.e., images) with the highest VI values were identified and the median value (VI_video_) was computed.


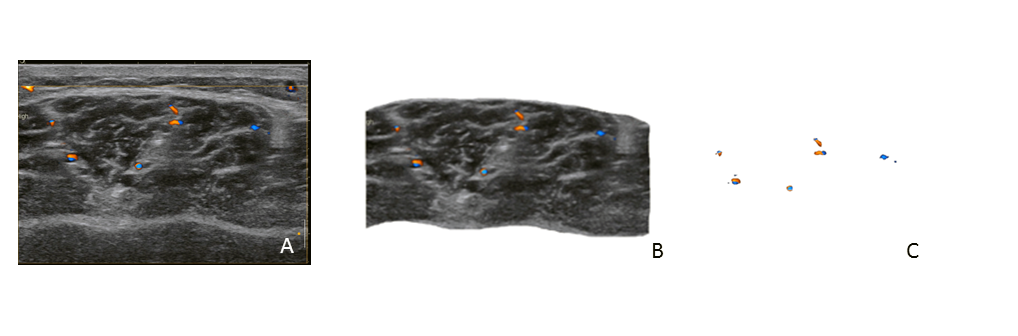
(A) A directional color power Doppler (dCPD) sonogram of the medial gastrocnemius muscle was captured in the transverse plane. (B) The border of the medial gastrocnemius muscle (i.e., the region of interest [ROI]) was manually outlined and then extracted by trimming the unwanted area from the outlined area. The total number of pixels within the ROI was counted by the algorithm. (C) The color pixels coded by the dCPD were extracted by eliminating the gray-scale pixels, and the number of color pixels was counted by the algorithm. The vascular index (VI) of the medial gastrocnemius muscle was calculated by the following equation: VI of the MG = (Number of color pixels within the ROI)/(Total number of pixels within the ROI). For this image, the VI value is 0.83%.

**Reference**

Ying M, Ng DK, Yung DM, Lee ES (2009) A semi-quantitative approach to compare high-sensitivity power Doppler sonography and conventional power Doppler sonography in the assessment of thyroid vascularity. Thyroid 19 (11):1265-1269. doi:10.1089/thy.2009.0221
